# Sustainable Lifecycle of Perforated Metal Materials

**DOI:** 10.3390/ma16083012

**Published:** 2023-04-11

**Authors:** Viktors Mironovs, Jekaterina Kuzmina, Dmitrijs Serdjuks, Yulia Usherenko, Mihails Lisicins

**Affiliations:** 1 Scientific Laboratory of Powder Materials, Faculty of Mechanical Engineering, Transport and Aeronautics, Riga Technical University, 6B Kipsalas Street, LV-1048 Riga, Latvia; viktors.mironovs@rtu.lv (V.M.); yuliausherenko@gmail.com (Y.U.); mihails.lisicins@gmail.com (M.L.); 2 Institute of Structural Engineering, Riga Technical University, 6A Kipsalas Street, LV-1048 Riga, Latvia; dmitrijs.serdjuks@rtu.lv

**Keywords:** perforated metal materials, PMM technological waste recycling, structural members, sustainable

## Abstract

In an era of rapidly growing consumer demand and the subsequent development of production, light materials and structures with a wide range of applications are becoming increasingly important in the field of construction and mechanical engineering, including aerospace engineering. At the same time, one of the trends is the use of perforated metal materials (PMMs). They are used as finishing, decorative and structural building materials. The main feature of PMMs is the presence of through holes of a given shape and size, which makes it possible to have low specific gravity; however, their tensile strength and rigidity can vary widely depending on the source material. In addition, PMMs have several properties that cannot be achieved with solid materials; for example, they can provide considerable noise reduction and partial light absorption, significantly reducing the weight of structures. They are also used for damping dynamic forces, filtering liquids and gases and shielding electromagnetic fields. For the perforation of strips and sheets, cold stamping methods are usually used, carried out on stamping presses, particularly using wide-tape production lines. Other methods of manufacturing PMMs are rapidly developing, for example, using liquid and laser cutting. An urgent but relatively new and little-studied problem is the recycling and further efficient use of PMMs, primarily such materials as stainless and high-strength steels, titanium, and aluminum alloys. The life cycle of PMMs can be prolonged because they can be repurposed for various applications such as constructing new buildings, designing elements, and producing additional products, making them more environmentally friendly. This work aimed to overview sustainable ways of PMM recycling, use or reuse, proposing different ecological methods and applications considering the types and properties of PMM technological waste. Moreover, the review is accompanied by graphical illustrations of real examples. PMM waste recycling methods that can prolong their lifecycle include construction technologies, powder metallurgy, permeable structures, etc. Several new technologies have been proposed and described for the sustainable application of products and structures based on perforated steel strips and profiles obtained from waste products during stamping. With more developers aiming for sustainability and buildings achieving higher levels of environmental performance, PMM provides significant environmental and aesthetic advantages.

## 1. Introduction

Worldwide annual resource use reached almost 90 billion metric tones in 2017 and could more than double by 2050 [1]. By 2030, European Raw Materials Alliance (ERMA) activities will increase the production of raw and advanced materials and address the circular economy by encouraging the recovered processing of essential raw materials [2]. In an era of rapidly growing consumer demand and the subsequent development of production, lightweight materials and structures with a wide range of applications are becoming increasingly important in the field of construction and mechanical engineering. Unfortunately, metal recycling tends to be at a low level globally. In 2010, the International Resource Group, hosted by the United Nations Environment Programme, issued reports on the metal stocks that exist in society and their recycling rates [3].

Perforated metal is a valuable source of secondary raw materials to be processed in metallurgical enterprises. The secondary use of perforated metal makes the process less expensive than conventional remelting methods, as well as significantly reducing emissions of harmful substances into the atmosphere and making it more sustainable as well as prolonging the lifecycle. There is an urgent need to change how we use non-renewable resources, especially metals, to maintain their sustainable availability and minimize the negative impacts associated with their production and use [4].

Products made of ferrous and non-ferrous metals or alloys are a valuable source of secondary raw materials to be processed in metallurgical enterprises. The use of scrap metal makes it possible to make the technological process of smelting metals less energy-intensive compared to obtaining products from ore and significantly reduces emissions of harmful substances into the atmosphere. Metals also can be returned to the production process without impairment. The steel sector will indeed be a key contributor to Europe achieving its 2050 targets. Thanks to their unique properties, ferrous and non-ferrous metals can be recycled indefinitely. The strong recycling performance is widely recognized and long-lasting. Moreover, the industry’s business model is circular by nature as the input of steel scrap is a necessary component for making new steel at any one of more than 500 steel plants in Europe. The more quality scrap that can be used in new steel production, the less raw materials and energy are needed and in turn, this reduces emissions. Steel, for instance, is circular by nature, because it is recycled repeatedly without loss of quality. Currently, of all steel packaging put on the market in Europe, 84% is being recycled into new steel products [5].

Solid waste generation is increasing in the world today. Zero-waste strategy is a smart solution to minimizing the increasing solid waste. To keep to a minimum solid waste, even more work is needed in the long run [6]. Reducing the resource intensity of products and emissions into the environment and improving their socio-economic performance throughout the life cycle of a material or product is an important task. Its solution can significantly facilitate the links between economic, social, and environmental aspects along the entire value chain of a product. At every stage of the life cycle, there is potential to reduce resource consumption and increase productivity.

Metals can be recycled back into the manufacturing process without compromising the quality of the subsequent product. In fact, recycling represents an advantage because it takes less energy to melt the metal. The recycling quota for scrap metal sent for recycling is 90% [7].

Modern architecture uses building materials in new and innovative ways. The fascination comes from the materials themselves and how they are processed and used. The emphasis is not so much on the material to be matched to the building, but on the effect of the particular materials created for the building with their material and visual qualities [5].

**Figure 1:**
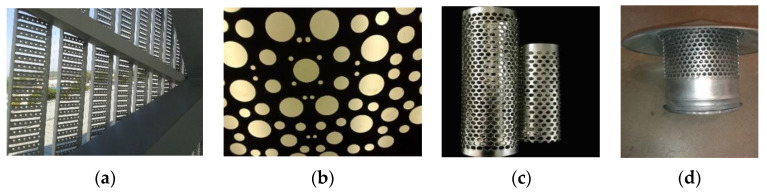
The use of PMMs: to reduce the level of illumination inside the room (**a**), increase the uniformity of the lighting of the elevator cabin (**b**), mute the sound (**c**) and ventilate the premises (**d**).

One of the trends in the manufacture of products and structures is the use of perforated metal materials (PMMs). These are used as finishing, decorative and structural building materials [8]. The growing interest is due to the fact that they significantly reduce the weight of the structure [9]. In addition, PMMs have a number of properties that cannot be achieved with solid materials; for example, they provide significant noise reduction [10], and light scattering [11]. They can also be used for damping dynamic force [12], filtering liquids and gases [13], and shielding electromagnetic fields [14]. PMMs are the simplest in terms of structure and implementation. They seem to be one of the most promising for wide production and application. The main feature of PMMs is the presence of through holes of a given shape and size. PMMs have low specific gravity, but their tensile strength and rigidity can vary widely depending on the source material [12]. This allows them to find a stable application, in particular in construction (Figure 1).

There are many examples of the effective use of PMMs in the creation of enclosing structures for buildings and mechanical structures [15], for the manufacture of facades and decorative elements, as well as in the production of fittings for pipes and panels. PMMs are increasingly used in various interior solutions for premises and urban architecture [16,17].

**Figure 2:**
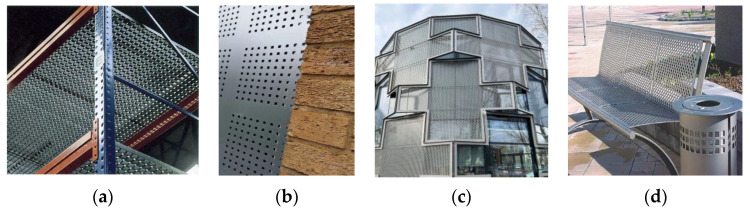
Structural units (**a**,**b**) and elements of building and garden furniture (**c**,**d**) from PMM.

In addition, PMMs have become popular and common materials for construction and installation works. Perforated steel strip components are widely used to connect and reinforce individual parts of building structures [16,17]. Most often, these are joints of load-bearing and enclosing structures made of drywall, as well as wood and concrete. In mechanical engineering, PMM products have found application in the manufacture of filters [18,19], separator assemblies, dryers, and other equipment [20] (Figure 2).

**Figure 3:**
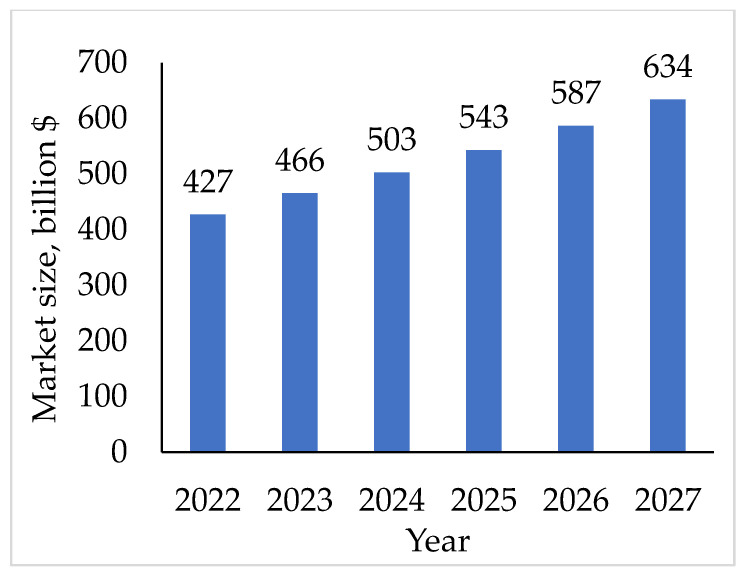
The estimated growth of the global stamped metal market [25].

For the perforation of strips and sheets, the cold stamping method is usually used, which is implemented on stamping presses, as well as by using broad tape production lines [21]. Other methods for the production of PMMs are rapidly developing, for example, using liquid [22,23] and laser cutting [24]. The volumes of production and use of thin-sheet stamping products in the world are constantly growing and the biggest sector is the expanding automotive market (Figure 3) [25].

**Figure 4:**
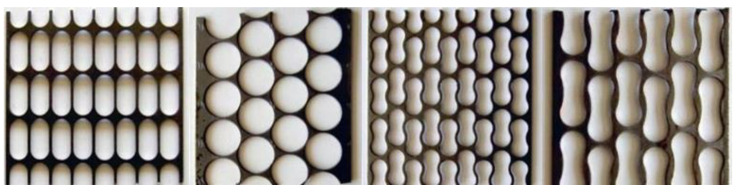
Some types of PMM residues with different shapes and sizes of hole samples.

The most common PMMs are steel, aluminum and copper. The main feature of PMM is the presence of through holes of a given shape and size (Figure 4).

In practice, the following physical and mechanical properties of PMMs are of the greatest interest: density, strength, ductility, weldability, corrosion resistance, hardness and microhardness and surface roughness [26,27]. When choosing materials for further use, an important point is the deformation state and the presence of corrosion phenomena. This is especially important when they are used in aggressive environments, such as marine, space, or aviation [28,29]. Experiments have shown that the tensile strength of steel perforated bands achieved as waste after punching is in the range of 100–250 MPa according to the type of perforation, width and thickness of the band and base material [30]. The percentage of perforation (%) of perforated bands, achieved after punching, varied from 66% to 76% when the thickness is in the range of 1.00 to 1.75 mm [30]. Studies have also shown that when perforations are made in the boundary layers, an increase in the microhardness of the material is observed and cracks may form. In particular, for steel, when stamping in the area of a punched hole (0.2–0.3 mm from the edge), the microhardness values almost doubled, but at a distance of 0.7–0.9 mm, it already reached the usual values for this material [31].

Punching or cutting perforation openings directly affects the mechanical and physical properties of the perforated metallic material. In many cases, the perforation process causes an increase in the surface hardness of the material, or forging [32]. During piercing or cutting, plastic deformation of the material occurs. Its degree on the edges of the perforation holes is very large and the grains of the metal microstructure are stretched in the direction of the applied load; therefore, their boundary surface can be invisible. In the process of forging the material, the specific volume of the metal increases and the density decreases, the ultimate strength, microhardness and brittleness increase while the plasticity and viscosity decrease. The stress concentration around the perforation opening increases. Even changes in the magnetic properties of the material are possible. Gradually moving away from the edges of the openings, the degree of metal deformation decreases. For high carbon steels, the zone of obvious deformation may be 0.3 to 0.4 mm from the edge of the perforation slots.

## 2. Technological Waste PMM and Methods for Their Further Processing

**Figure 5:**
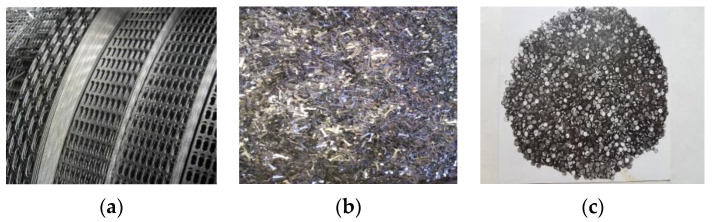
PMM waste: rolls of steel tape (**a**); after rough grinding (**b**); in the form of pistons (**c**).

PMM waste is generated during the reconstruction or demolition of buildings and structures in which they were used [33]. Another significant type of PMM technological waste is waste after stamping parts from a sheet or from a strip (cutting) (Figure 5).

### 2.1. PMM Waste in Construction Technologies

Due to its diverse geometric parameters, physical and mechanical properties, PMM technological waste, primarily from steel and aluminum, can be effectively used in construction in the manufacture of enclosing structures, elements of facades and interiors, lighting devices, and the manufacture of some construction and finishing tools [34,35]. PMM can be used for producing several types of load-carrying, insulating and decorative structural members [17,36].

**Figure 6:**
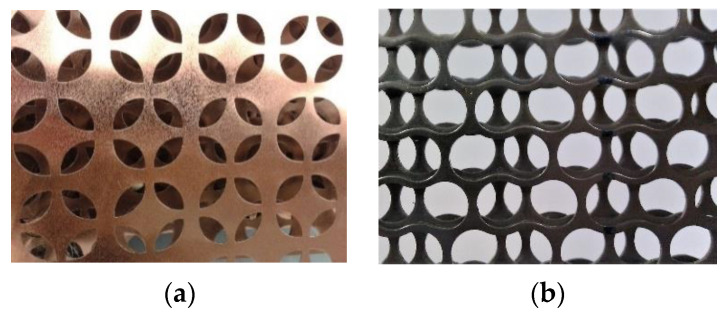
Fragments of panels: from two perforated steel strips laid with a copper shift (**a**), from steel several strips laid in parallel (**b**).

The main functions of building envelopes can be called thermal insulation and protection from negative external factors. Enclosing structures can consist of either one layer or several, for example, the outer cladding and the inner part, as well as insulation between them [37]. When we use the overlay method of perforated sheets or strips, we can obtain perforated frames. The imposition of PMMs can be multi-layered, which provides a more stable structure while maintaining the overall dimensions and creating a new ornament (Figure 6).

The connection of perforated sheets or strips is easily achieved by welding [38], soldering, bolting, or gluing [39]. The ability to overlap sheets in both parallel and perpendicular directions, adjust the mutual displacement of the perforation, and combine their sizes, allows us to significantly diversify the shapes of the resulting panels and their properties.

**Figure 7:**
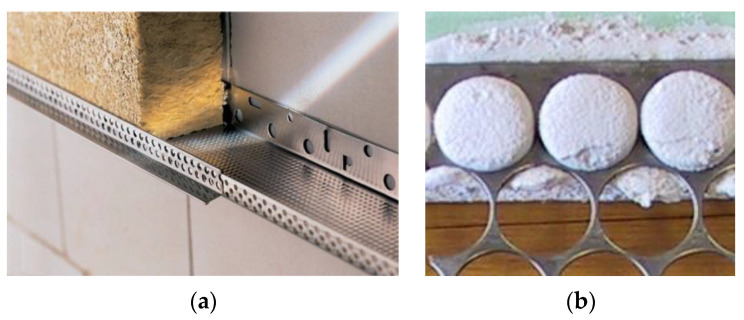
The solution for the partition wall construction using perforated steel profiles: (**a**) structural solution of self-bearing insulation panel with the framework of PMM-made perforated steel profiles, (**b**) plasterboard and perforated steel tape connecting with gypsum-based glue [41].

PMM-made perforated steel profiles can also be used for wall frames or structures as stiffening elements. Currently, self-bearing partitions and external wall finishes are often used for non-perforated metal profiles. One of the ways of creating the framework and providing the necessary stiffness for insulation wall panels is the use of perforated double-T profiles. In this way, the wall frame is considerably lightened [40]. The profile cavities are filled with insulation or soundproofing material inserted into the profiles, and the structure is insulated from the outside with plasterboard sheets or other finishing material. The perforation of the profiles provides opportunity for the plasterboard to connect it by fast-setting adhesive, such as gypsum-based glue. A very simple and effective wall structure, which contains heat and sound insulation, and the supporting frame of the wall is made of PMM in the form of a corner or channel (Figure 7).

The jointing of plasterboard sheets with perforated profiles is simple and has high productivity, which is why this method is recommended for self-bearing, quick-to-install construction. Plasterboard partition walls, using PMM and gypsum-based adhesive as the bonding element can be 20-27 cheaper than the traditional fixing option with tin profiles and screws [41].

PMM can also be used as a raw material for metal or composite sandwich panels where PMM forms the core of the panel and can also be used for upper or lower flanges. The principle of sandwich panels is similar to that of a double-T beam. In bending, the panel’s top flange is compressed while the bottom flange is tensioned. The cell structure core analogs the beam wall, accommodates shear stresses, increases the stiffness of the structure and provides a certain distance between the flanges [15]. Compared to a beam wall, the panel core has the advantage of supporting the flanges over the entire surface area, forming a uniform reinforced structure. The separation of the flanges increases the moment of inertia of the panel cross-section by a slight increase in weight. This results in an efficient structure with good bending strength and stability. The use of perforated sheets also provides additional options for the fixing of the panels, depending on the structural solution [42,43].

**Figure 8:**
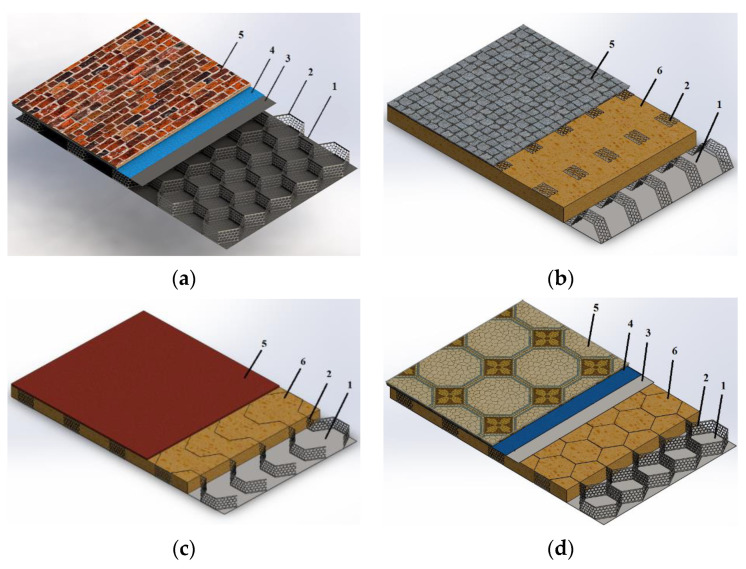
Sandwich panels with the PMM core, (**a**) external panel without heat insulation: (**b**,**c**) internal panel with heat or sound insulation; (**d**) ceiling panel; 1—bottom flange; 2—perforated profile; 3—load distribution layer; 4—damping layer or adhesive layer; 5—finishing layer; 6—heat or sound insulation [44].

Using PMM, it is possible to produce various honeycomb structures. Several sandwich panel solutions based on perforated steel strip cores are shown in Figure 8.

**Figure 9:**
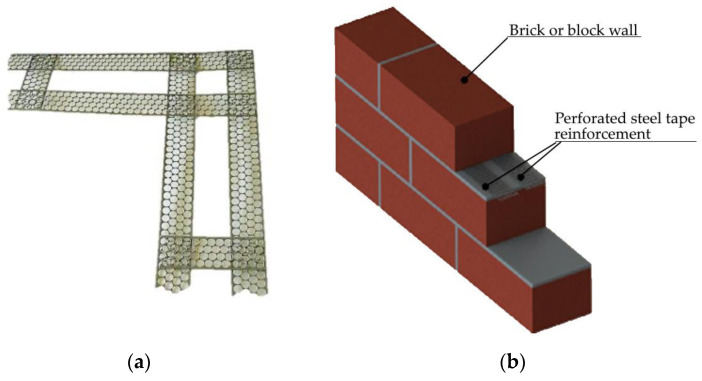

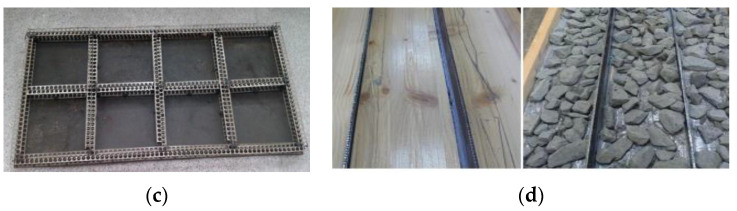
PMMs use different structural technologies: (**a**,**b**) PMM in masonry, (**c**) stiffeners in the mould members, (**d**) members providing timber-to-concrete connections in timber–concrete floor panels.

The panel constructions [44] shown in Figure 8 differ mainly in profile type and their arrangement. The profiles can be based on perforated steel of different widths and steel tapes of different thicknesses. The proportion of perforation may also vary [41]. The profiling of the tapes may differ, depending on the structural design of the panel and the expected load. The profiles may be arranged in both planes about the flange (Figure 9b) or upright (Figure 9a,c,d). All of these parameters are chosen considering the mechanical properties of the profile materials and the final product [45].

PMMs can also be effectively used as auxiliary elements in various construction technology processes, such as concrete pouring. There are some research and practical applications of PMMs in cement composite thin wall structures, where their use is of structural and operational efficiency but the labor intensity of such structures can also be a challenge that needs to be addressed. [43,46].

The application of PMM for timber-to-concrete connections in floor panels enables us to increase by 19% its load-carrying capacity panels. It has been stated that the existence of the holes has a significant influence on the load-carrying capacity of timber–concrete floor panels. The increased sustainability is provided by the 20% replacement of the metal materials by the PMM waste. [40]. It was stated, that existence of the holes has a significant influence on the load-carrying capacity of the timber–concrete floor. The use of steel PMM makes it possible to improve the quality of the brickwork by obtaining a thinner cement joint in the masonry, to increase the adhesion of metal to cement mortar. (Figure 9).

Another one of the possible directions for the use of tape PMMs is the production of armor-reinforced products for hydrotechnical construction [44].

### 2.2. Use of PMM Waste in Powder Metallurgy

**Figure 10:**
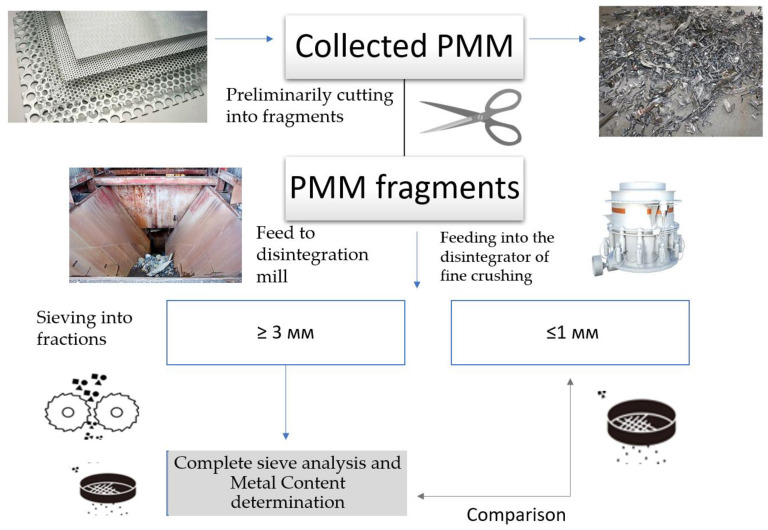
Scheme of processing PMM waste into powder by mechanical grinding.

With large volumes of PMM waste, it is advisable to consider the issue of their use as a raw material to produce powders. As an experiment, we carried out research on the grinding into powder of belt waste generated during the manufacture of drive chain elements [12,20]. To carry out research on the possibility of using PMM waste in powder metallurgy, a perforated tape made of steel 50 was used, which is a technological waste after stamping elements of drive chains. Grinding was carried out in several stages: cutting a sheet roll into fragments, coarse and fine crushing, and grinding the material into powder (Figure 10).

**Figure 11:**
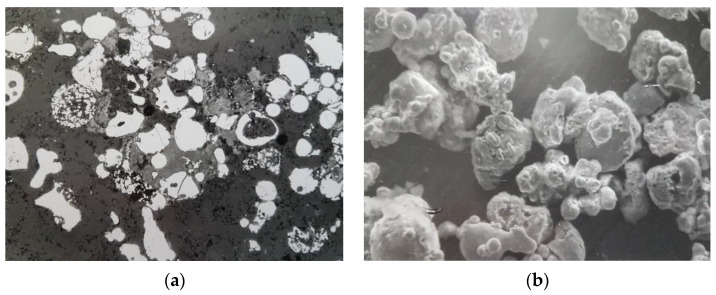
Microstructure of powder from steel 50 after mechanical grinding (**a**); obtained by melt spraying (**b**).

The disadvantage of the method is increased energy consumption and an increased percentage of pollution (Figure 11a).

Another method we used—obtaining a powder by melt spraying—allows for obtaining powders of higher quality (Figure 11b).

### 2.3. PMM Waste for the Manufacture of Frames of Technological Equipment and Devices

**Figure 12:**
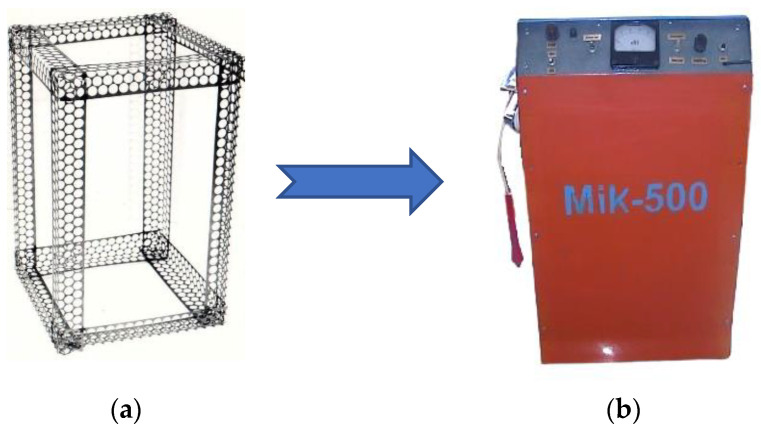
Prismatic frame made of angle-type steel PP (**a**), the device with steel sheet cladding (**b**) [44].

For the manufacture of frames, waste tape from PMM is most suitable, from which box-shaped profiles (PP) are pre-made. They have a number of advantages: low weight, ease of fastening sheet sheathing, etc. The example of a prismatic frame made of angle-type steel PP to obtain the device with steel sheet cladding Is shown in Figure 12.

**Figure 13:**
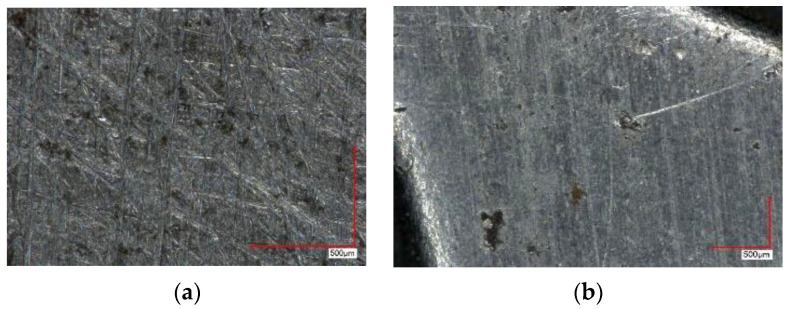
PMM microstructure with traces of corrosion: they can be superficial (**a**) and deeper (**b**).

Used materials, as a rule, have surface damage–corrosion areas, non-linearity and other defects (Figure 13).

The growth of emerging markets increases the importance of a local presence [47]. That is why the reuse of perforated metals for architecture and design finds actuality in the market. As an example, decorative permeable and non-permeable lightweight grids, shields and wall structures can be developed. The grids can be partly covered with a polymeric material–rubber or plastic to produce reinforced decorative elements [45], which can simultaneously serve as delimiting and separating shields.

A protective coating can be applied to the fabricated grid, and the grid is then installed in a wooden or metal frame. In this way, the design becomes more attractive. Such types of elements can be both walls or hatches, delimiting different rooms, but allowing light to flow through. PMM can be successfully used in the design of lighting devices, in particular, in the manufacture of lamps for outdoor lighting. In this case, the decorative body is made from perforated steel strips obtained from stamping waste. Corps is running in the form of a sphere, cylinder or cone. To achieve this, you can use the processes of profiling and welding PMM The proposed solution allows us to reduce the weight of the lighting. device, reduce the cost of its production, as well as expand the scope of application by increasing and diversifying the luminous flux [48].

### 2.4. Permeable Structures from PMM Waste

**Figure 14:**
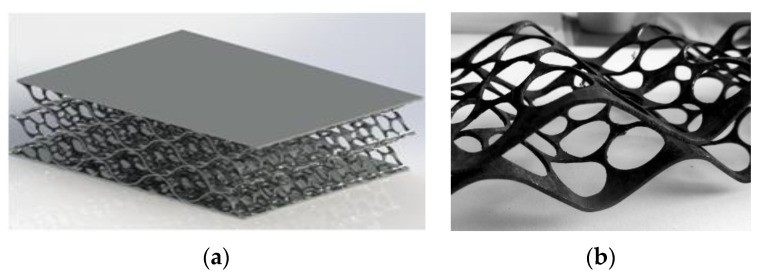
Lattice sandwich panel (**a**), using several perforated profiles (**b**).

A promising area of application of PMM frames is the creation of panels for absorbing mechanical energy. This refers to mechanical collision with moving bodies or vibration caused by the operation of the equipment. The core of the panels in this case should be of sufficient strength and rigidity, but at the same time sufficiently easily compressible. Its profile should be flat in relation to the facing sheets. Cellular hexagonal and lattice structures were used as cores for such panels in (Figure 14). This is achieved by layer-by-layer laying of PMM profiles. In this case, additional materials from polymers can be used.

The amount of absorbed energy depends to a large extent on the mechanical properties of the facing sheet materials, the material and the thickness of the panel core. Studies have shown that it is possible to form a panel from several profile layers and intermediate cladding layers. The thickness of the material naturally increases in this case. Therefore, the required energy absorption capacity of the panels must be carefully evaluated. In addition, increasing the thickness also increases material consumption and overall costs [44].

Various elements with a permeable structure can be made from waste sheet and tape materials, which are effectively used in the construction of various types of ventilation and filtering equipment, as well as in sound-absorbing devices [49]. Such structures made from PMM waste can be single-layer or multi-layer. The size and location of the perforations can also vary.

**Figure 15:**
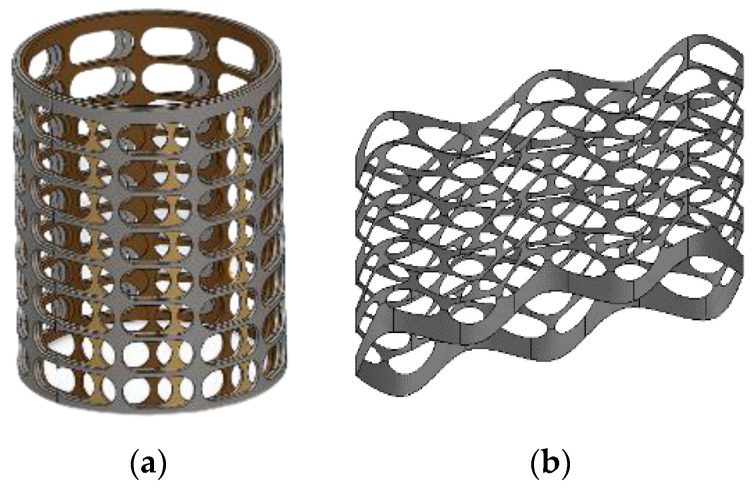
Products with a permeable structure: cylindrical shape(s), obtained by folding a tape or sheet and welding to the height of the cylinder(s) (**a**); with complex spatial configuration, obtained by profiling tapes and connecting them by spot welding (**b**).

Figure 15a shows an example of a structure consisting of several cylinders of different diameters and perforations of the same shape with the possibility of displacement of one cylinder relative to another. Such a design, made from waste steel perforated tape, can be effectively used for drying materials and products [15]. Active ventilation is provided by air supply through the perforation. Drying channels made of PMM can be made in the form of semi-cylinders. In this case, they are easy to install and place in the factory yard or warehouse and easy to assemble and transport. The spatial structure can be made from tape PM using bending and welding processes (Figure 15b).

**Figure 16:**
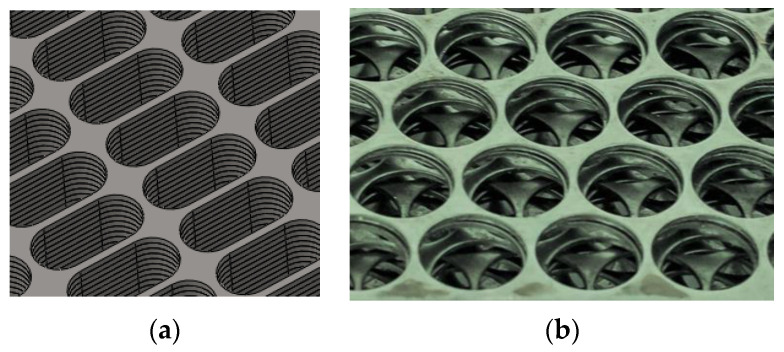
Formation of flow channels in permeable structures: by the layer-by-layer laying of the tape without mutual displacement of layers (**a**); with mutual displacement of cell structure laminar airflow (**b**).

It is expedient to use cellular structures from PMM for the manufacture of deflectors, elements of refrigeration equipment, and climatic chambers. In [50], we proposed to fabricate metal permeable structures by layer-by-layer coating. The length and direction of the flow channels are adjusted by mutual displacement of the perforated sheets (Figure 16). Hexagonal mesh deflectors provide high flow efficiency, eliminate turbulence, fouling and moisture transfer, and reduce noise and energy consumption [10].

The permeable design allows the flow of liquid or gas to be controlled over time. This is achieved by mutual displacement of the perforated elements and by filling the created channels with filter material.

**Figure 17:**
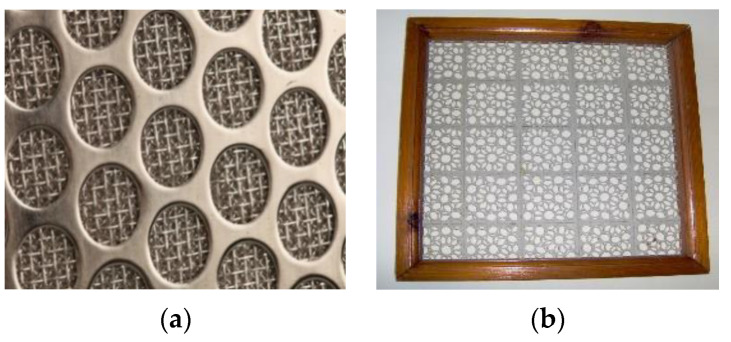
Mesh filter in a perforated steel frame (**a**) and wood frame (**b**) [44].

Perforated elements can be fixed in specially made cases or frames (Figure 17). In this case, PMM is used both as the filter material itself (usually this is the first stage of the filter), and as a frame for the internal filter.

### 2.5. Metal Screens from the Waste of Perforated Sheets

A number of works have shown that perforated metal screens (PMM made from tape or sheet waste can be successfully used to protect against light, sound, and electromagnetic radiation [51,52].

**Figure 18:**
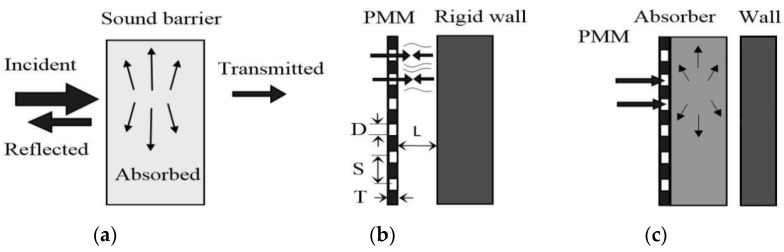
General schemes of sound absorption PMM: sound transformation scheme (**a**), absorption scheme using a protective perforated wall (**b**). also, with the addition of soundproofing material(s) (**c**) (D—diameter of holes, S—distance between hole centers, T—screen thickness, L—distance from screen to object surface).

The presence of perforations allows some of the sound waves to penetrate inside and activate the absorption mechanisms. In the case of PMMs, there are two. The first one is the damping of sound energy during the passage of a wave through perforated holes due to frictional losses of oscillating air molecules interacting with the walls of the hole. Micro-perforated PMMs, in which the perforations are reduced to millimeter or sub-millimeter sizes, provide very effective sound absorption without any additional classic absorbing material [53]. Micro-perforated sheets are of particular interest to the aerospace and aircraft industries, as well as in various industrial cases [50]. The second mechanism is the Helmholtz resonator. In this case, a rigid wall reflecting sound waves is placed behind the PMM sheet at a distance L (Figure 18). Absorption occurs due to oscillations of the air column enclosed between the wall and the hole. To give the barriers maximum sound insulation, additional absorbers made of porous or woven materials with a thickness of 2–10 cm are provided in the design.

**Figure 19:**
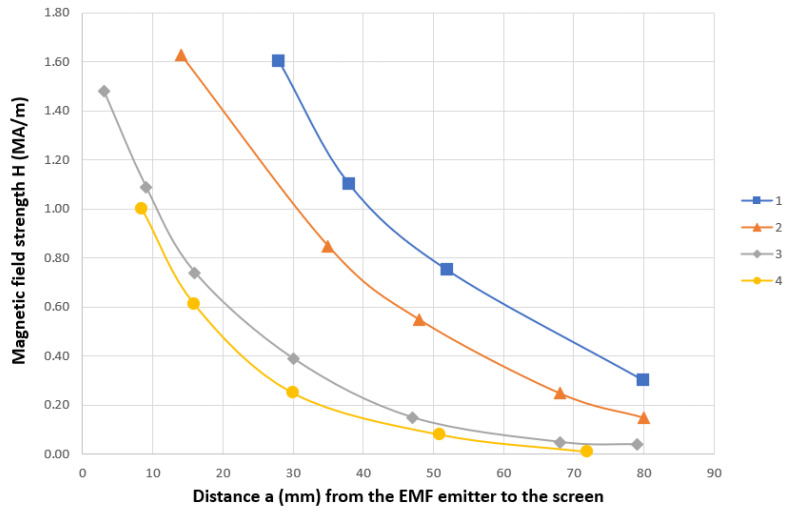
Damping of pulsed EMF in the axial and transversal position of the screen shielding: 1—axially to magnetic flux without shielding screen; 2—axially to magnetic flux shielding screen; 3—transversally to magnetic flux without shielding screen, 4—transversally to magnetic flux shielding screen.

Screens for protection against electromagnetic fields can be made from PMM waste. This experiment was considered in the work [54]. An experimental analysis of the screening efficiency of protective screens made of perforated steel tapes was carried out (Figure 19). It has been established that perforated screens reduce the intensity of the pulsed electromagnetic field (frequency 30 kHz) to 20–30%. However, the effectiveness of shielding materials depends on many parameters and must be evaluated for each specific application.

Research in the field of passive protection of spacecraft structures has shown a growing demand for lightweight and economical materials that are needed to counteract the damaging effects of micrometeorite orbital debris [55].

The speed of collision of space vehicles with meteoroid bodies and space debris is in the range of 1–50 km s^−1^. At such impact velocities, intense energy release occurs in a limited volume of matter, accompanied by the formation of shock waves, followed by mechanical destruction, melting, evaporation, and thermal ionization of the resulting vapors [56].

At the above-mentioned impact velocities, particles with a cross-section size of more than 0.5–1 cm can create penetrations in spacecraft walls and lead to catastrophic destructions. Impacts of small particles on the surface of a spacecraft cause the formation of craters and scratches on the surface, and in the case of a large number of impacts—noticeable erosion of the surface. Various optical elements suffer the most from small particle impacts: portholes, lenses, safety glasses, mirrors, etc. Plasma emissions and light flashes resulting from strong heating of the substance in the high-velocity impact zone can have a negative effect on sensors of scientific equipment and some units of spacecraft electrical and radio equipment [57].

**Figure 20:**
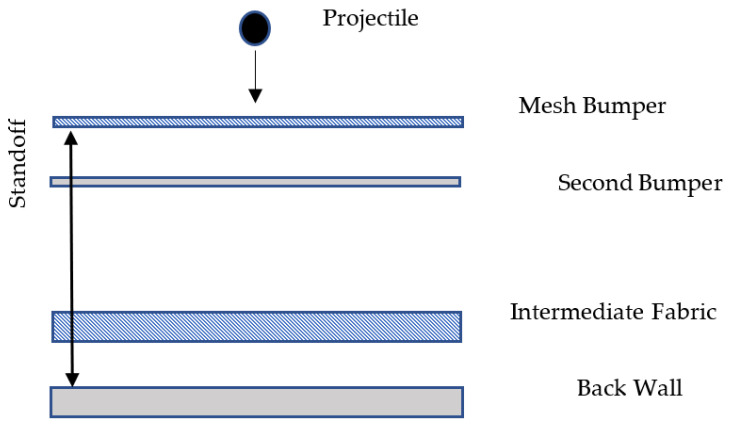
Mesh Double Bumper shield.

A variety of structural materials, both homogeneous and composite, can be used to produce shields. The shield design can be either single- or multi-layer. Hypervelocity impact research led to the development of numerous shield configurations, such as the Whipple, Stuffed Whipple, metallic foam sandwich Whipple, Multi-Shock, and Mesh Double Bumper shields (Figure 20).

The study of metal mesh shields was carried out in [58,59]. It showed that they possessed higher efficiency than solid shields. It was found in the research [60,61,62] that the resistance of such screens can be increased due to various geometric features of their components (mutual arrangement of layers, angle of inclination, etc.), as well as from the materials of the layers and their order.

**Figure 21:**
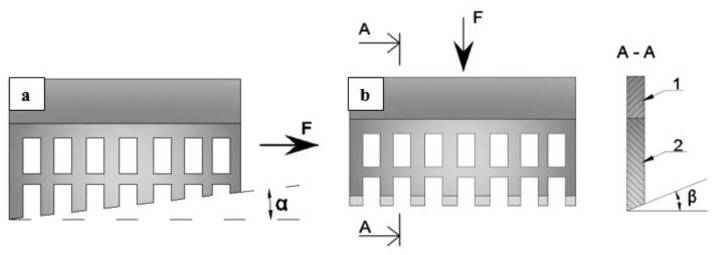
Single-layer cutting element with inclined teeth made of perforated steel strip: for horizontal (**a**) and vertical cutting (**b**) (F—pulling force, 1—gripper, 2—cutting blade).

It is promising to use strip perforated steel materials in the form of cutting comb elements for the manufacture of machining tools. This proposal refers to the field of mechanical engineering, but can also be used in the construction industry, for example, in the manufacture of tools for finishing work (Figure 21). The shape and design of the cutting elements may vary depending on the intended use. Thus, for example, Figure 21a shows a cutting element consisting of a single perforated steel strip with cut and sharpened teeth. It is designed for cutting in the horizontal direction. In the second case (Figure 18b), the tool is designed to cut materials in the vertical direction [48].

## 3. Conclusions

The work showed sustainable ways to recycle or reuse perforated metal materials. Various new technologies are proposed and described for the sustainable application of PMMs. Reviewed methods are more environmentally friendly and they demonstrate a clear possibility of prolonging the lifecycle of PMMs through their reuse in numerous applications, thereby conserving resources and energy. The zero-waste orientation of PMMs makes them a smart way to minimize waste and attain the European Union’s sustainable goals of becoming climate-neutral. PMMs provide significant environmental and aesthetic advantages, which align with the circular economy’s principles of a closed-loop system and waste minimization.

The types and properties of PMM wastes were considered and investigated. It was stated that for small volumes, PMM waste can be used in load-carrying, insulating and decorative structural elements, for producing sound-protective screens, as well as frameworks and stiffeners for the casings of various devices. Moreover, the previous use of PMM waste in producing instrument frames, protective screens, and building fragments suggests that further investigation is warranted. Using strengthening elements made of PMM wastes increases the load-carrying capacity, stiffness and safety of timber, concrete, and masonry structural members. For example, the application of PMM for timber-to-concrete connections in floor panels enables its load-carrying capacity to be increased by 19%.

With large volumes of waste in the form of die-cutting, a sustainable lifecycle can be achieved by processing it into metal powder for the purposes of metallurgy. An important role is played by the processes of cutting, welding, bending and profiling of sheet and strip waste. Particular attention should be paid to assessing the general deformation state and especially the corrosion of perforated materials.

## Data Availability

Data supporting the results presented can be provided upon request to the respective author.
